# Monocyte and Macrophage miRNA: Potent Biomarker and Target for Host-Directed Therapy for Tuberculosis

**DOI:** 10.3389/fimmu.2021.667206

**Published:** 2021-06-25

**Authors:** Pavithra Sampath, Krisna Moorthi Periyasamy, Uma Devi Ranganathan, Ramalingam Bethunaickan

**Affiliations:** Department of Immunology, National Institute for Research in Tuberculosis, Chennai, India

**Keywords:** monocyte and macrophage miRNAs, tuberculosis, differential expression, immune regulation, autophagy and biomarkers

## Abstract

The end TB strategy reinforces the essentiality of readily accessible biomarkers for early tuberculosis diagnosis. Exploration of microRNA (miRNA) and pathway analysis opens an avenue for the discovery of possible therapeutic targets. miRNA is a small, non-coding oligonucleotide characterized by the mechanism of gene regulation, transcription, and immunomodulation. Studies on miRNA define their importance as an immune marker for active disease progression and as an immunomodulator for innate mechanisms, such as apoptosis and autophagy. Monocyte research is highly advancing toward TB pathogenesis and biomarker efficiency because of its innate and adaptive response connectivity. The combination of monocytes/macrophages and their relative miRNA expression furnish newer insight on the unresolved mechanism for Mycobacterium survival, exploitation of host defense, latent infection, and disease resistance. This review deals with miRNA from monocytes, their relative expression in different disease stages of TB, multiple gene regulating mechanisms in shaping immunity against tuberculosis, and their functionality as biomarker and host-mediated therapeutics. Future collaborative efforts involving multidisciplinary approach in various ethnic population with multiple factors (age, gender, mycobacterial strain, disease stage, other chronic lung infections, and inflammatory disease criteria) on these short miRNAs from body fluids and cells could predict the valuable miRNA biosignature network as a potent tool for biomarkers and host-directed therapy.

## Introduction

Tuberculosis being the life-threatening disease caused by *Mycobacterium tuberculosis* (MTB) is intricate to understand their mycobacterial-mediated host immune subversion. The intracellular nature and delayed cell division of MTB added access to dodge the host microbicidal effect for its survival. The host’s innate defense ability and the pathogen’s strategy in evading the host’s immunity determine the sequel of TB infection (1). MTB establishes infection through multiple modalities, such as i) circumvent phagolysosome fusion and phagocytosis destruction; ii) neutralize the acidic environment (2, 3); iii) blocks the formation of the apoptotic envelope (4); iv) inhibits the plasma membrane repair, leading to the spread of infection through macrophage necrosis (5); v) suppresses activation of immune cells and antigen presentation; vi) limits the proinflammatory response by restricting proinflammatory cytokines; and vii) modulates the disease responsive genes and miRNAs through their targeted pathways. The disease becomes complex as the stages of infection are varied from latency to drug resistance because of the evolution of MTB strains. One third of the population exhibit latent infection, in which MTB remains dormant for a long period and becomes susceptible to the active disease under immune compromised condition. This latency is a menace to mankind as the diagnosis and its effective treatment toward breakdown of the disease in future need unbridled enthusiastic investigations. However, the management of the latent condition can be made possible with public awareness by improving the incidence of TB determinants, such as malnutrition, poverty, smoking, and diabetes, or through the development of new treatment or vaccines (6). The emergence of drug-resistant Mycobacterium due to poor treatment adherence (acquired resistance) and the transmission of drug-resistant strains (primary resistance) is another peril in TB research toward the end TB strategy (7). The multi-drug resistance and its treatment pose multiple challenges as it requires prolonged treatment duration, complex drugs (second-line fluoroquinolones) that may affect adherence along with lower treatment success rate (6). Other co-morbidities, like AIDS and diabetes, intensify TB disease pathogenesis.

Mononuclear cells (monocytes/macrophages) are professional phagocytic defenders against TB infection (8). The disputed behavior of monocytes as a defender against antimycobacterial activity exhibited by CD16^neg^ subset and habitat for MTB promoted by CD16^pos^ subset is well accepted for TB disease (9, 10). The disease-specific perturbation in the mononuclear cell subsets and their immune phenotypes contributed to underlying pathophysiology and as biomarkers for MTB infection. However, the unresolved mechanisms and the pathways affected can be studied through the molecular impression of these subsets from omics platforms in a quest for differentially expressed mRNAs and miRNAs. miRNAs are short, biologically conserved noncoding RNAs that participate in the regulation of inflammatory response, tumorigenesis, and other biological processes. Several studies focused on miRNAs revealed altered miRNA levels during infection and their impact in modulating immune functions within macrophages from TB patients (11–13). Thus, miRNA studies open up new avenues and fascinate the researchers for constructing miRNA-based vaccines, biomarkers, and host-directed therapies. This review is focused on monocyte/macrophage miRNAs, their differential expression, regulatory function, and biomarker utility in tuberculosis disease.

## miRNAs

Micro RNAs are discovered as biologically conserved, short noncoding RNAs (14–16) that constitute 18 to 25 nucleotides in length. This groundbreaking innovation by Ambros and Ruvkun prompted the researchers to investigate their functional behavior toward host immune regulation and disease pathogenesis, which resulted in the exponential growth of published studies on miRNA reported by Almeida et al. (17).

miRNAs work as mRNA repressors inhibiting protein synthesis (18), translational activators (19), and molecular decoys for RNA-binding proteins (20), depending on the environment and cell type. The processing, maturation, expression, and action of miRNAs are regulated through multiple mechanisms: a) single-nucleotide polymorphism interfere with the processing and maturation of miRNAs that affect their expression profile (21); b) modulation of epigenetic mechanisms, such as histone acetylation and DNA methylation, influence the transcriptional rate of miRNAs (22); c) impairment in the mRNA-miRNA interactions by the competition of miRNAs with cellular factors and mRNAs with other competitive RNAs (pseudogenes, long non-coding RNAs, and circular RNAs) (23, 24); and d) occurrence of miRNA editing through nucleotide modification by adenosine or cytidine deaminases (21, 25). miRNA research and transcriptomic platform enabled the disease-mediated deregulation of miRNAs and their targeted pathways in multiple diseases, including cancer (26, 27), cardiovascular diseases (28, 29), autoimmune diseases (30, 31), and infectious diseases (32, 33).

## Monocyte and Macrophage miRNAs

The disease-oriented modification for any microbial infection is visualized primarily on monocytic cell lineage as being the first-line defenders of innate immunity. Immunological aspect-derived alterations in the subset composition of monocytes/macrophages decipher the role of a pathogen in the peripheral compartment. However, the stimulus for the alteration is better studied through their responsive mRNA and miRNAs. miRNA research for TB is advancing toward a proper understanding of disease mechanism for better prognosis and early prevention. The immune efficiency and other cellular processes of monocyte/macrophages are governed by various miRNAs in both healthy and disease states (34).

Many reports available for the miRNAs mediated monocytic biological functions, such as tissue homeostasis, signaling, cell differentiation, apoptosis, cell motility, cytokine production, inflammatory responses, resolution of inflammation, and other immune responses (35–40). A trio of miRNAs constituting miR-146a, miR-21, and miR-155 are the principal regulators of inflammatory pathways in myeloid cells (41). miR-511 was identified as the putative positive regulator of Toll-like receptor 4 during monocyte differentiation by Tserel et al. (42). miR-214, as suggested by Li et al., targets the phosphatase and tensin homolog in monocyte survival induction during advanced glycation (43). miR-20a, miR-106a, and miR-17 of miR-17/92 and miR-106a/363 clusters are involved in tuning the proinflammatory cytokine production, infiltration of macrophages, and phagocytosis through targeting the expression of signal-regulatory protein alpha (44). Upon Notch activation, miR-148a-3p promotes M1 polarization by hindering M2 activation (45). Myeloid cell differentiation to granulocytes or monocytes is governed by miR-223 with negative control on NLRP3 inflammasome activity (46).

The intense research on miRNA profiling of monocyte subsets delivered their unique profile and regulated functions. Dang et al. deciphered the role of miR-432 in apoptotic potential and miR-19a in cell motility. They also observed that miR-345 was involved in the inflammatory responses by targeting RelA. Besides, upregulated miR-34 in CD16+ monocytes are suggestive of their differentiation ability to dendritic cells by altering the expression of Wingless-Type MMTV Integration Site Family, Member 1 (WNT1), and Jagged 1 (JAG1) (34, 47). Richard et al. focused on the sequencing of miRNAs among monocyte subsets in humans and mice to identify their role in monocyte heterogeneity. From their work, they suggested three miRNAs—miR-21, miR-150, and miR-146a—as immune regulators that mediate resolution of inflammation in the myeloid cells (48). MicroRNA profiling of intermediate monocytes (CD14++ CD16+) yielded a unique miRNA profile, and their connected pathways are involved in gene regulation, TLR, and cytokine-mediated signaling, phagocytosis, antigen processing, and presentation, as well as lipid and triglyceride metabolism (49).

## MicroRNA as a Prominent Immune Regulator of Macrophage Mechanisms During TB

**Figure 1:**
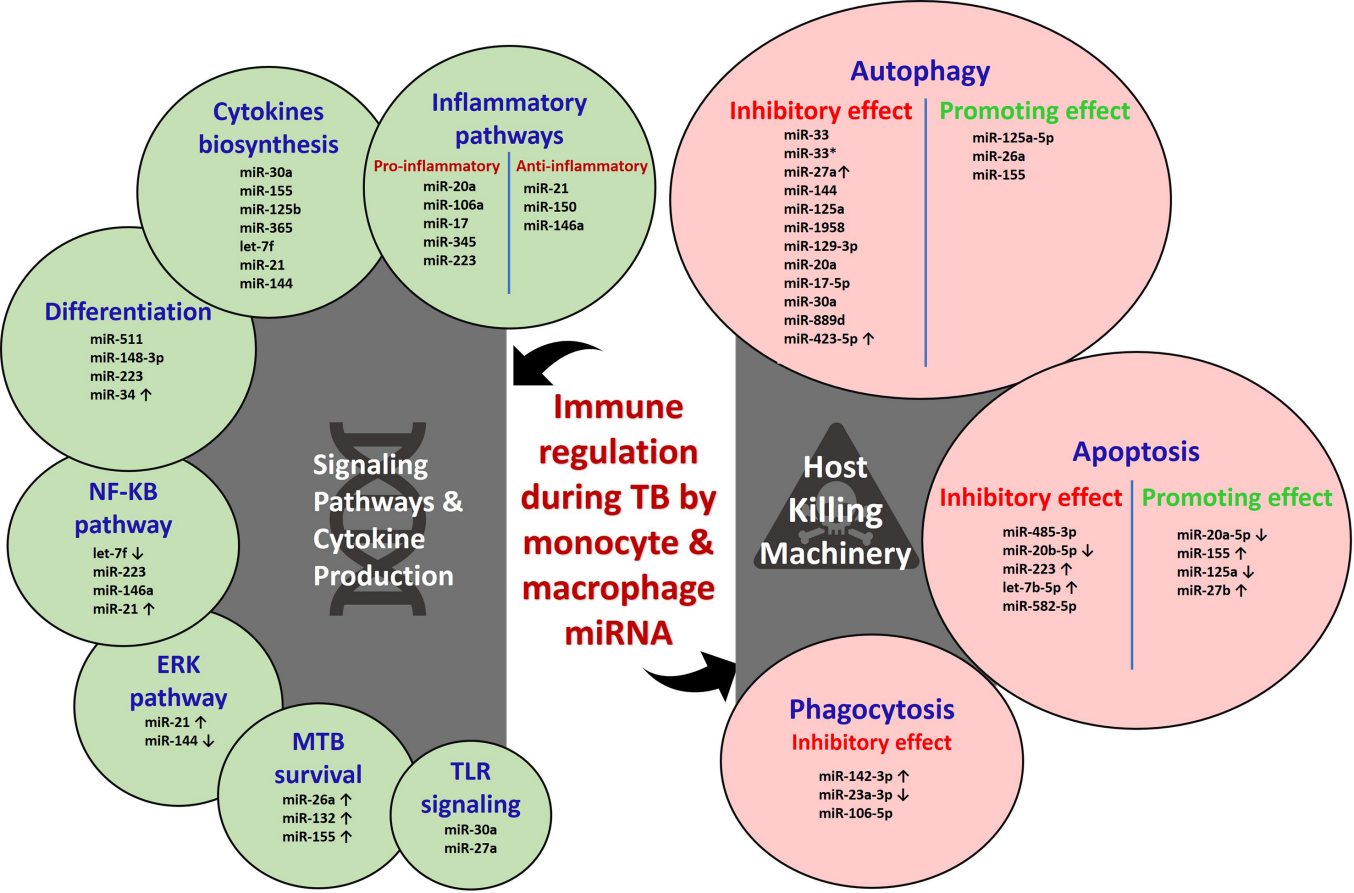
Host immune regulation by monocyte and macrophage miRNAs during tuberculosis.

miRNAs regulate about 60% of mammalian genes through its effective binding to 3′ UTR on mRNA and leads to translational repression and mRNA degradation (50, 51). Most of the cellular functions in humans are governed by single or multiple miRNAs. The emergence of miRNA research uncovered the possibility of pathogen (specially their cell wall components) induced alteration of miRNA levels (52). The altered miRNA profile could enhance the disease progression by modulation of the innate and adaptive responses through the hindrance of cell differentiation (53). The distinctive role of miRNA in the maintenance of immune homeostasis and activation of immune defense is largely studied (54). Upon MTB infection, several miRNAs modulate the host mechanism, either favoring the host or the pathogen. In most cases, the underlying causes for host immune evasion by the Mycobacterium are associated with miRNAs. The host signaling pathways, cytokine production, and killing machinery are adversely affected by miRNAs as represented in **Figure 1**.

## miRNAs in Signaling Pathways and Cytokine Production

The prime innate defense recognition starts with the Toll-like receptors (TLRs) upon induction with pathogen-associated molecular patterns (PAMPs). However, this initial priming is affected by multiple miRNAs during MTB infection. TLR/MyD88 activation and cytokine response are inhibited by miR-30a in MTB-infected THP-1 cells (55). TLR3 signaling is attenuated by miR-27a through targeting TICAM1 and c-Abl-BMP signaling (56). Survival of Mycobacterium is favored through the upregulation of miR-26a and miR-132 induced by live and attenuated MTB that negatively controls p300 mRNA in human monocyte-derived macrophages (human MDMs). miR-132 and miR-26a dampen the host responsiveness toward IFN-gamma genes, phagocytosis process, and decreases the HLA-DR and FCgammaR1 levels (57). Inhibition of NF-κB pathway with the hindered downstream secretion of cytokines, chemokines, and NOS is achieved through the increased expression of A20 (TNFAIP3) by downregulated let-7f induction mediated by ESAT-6 in both *in vitro* and *in vivo* conditions (58). miR-223 and miR-146a also negatively control the NF-κB pathway in MTB-infected macrophages and suppress the proinflammatory response and the clearance of pathogen (59–62). Infection with BCG induces elevation of miR-21 *via* NF-κB and ERK pathways that target IL-12p35 mRNA through which it inhibits IL-12 production and T-cell priming function by APCs (63).

The activity of miR-155 is focused on various cell types, such as macrophages, dendritic cells, and T cells. ESAT-6 induces miR-155 in a time- and dose-dependent manner, which downregulates SHIP1, leading to an ultimate increase of the AKT phosphorylation and, thus, exerts pro-survival of MTB on macrophages. Host IL-6 production and Cox-2 activity are limited by upregulated miR-155, as the Cox-2 is essential to prevent necrosis by generating PGE_2_ and restricting lipoxin A_4_ (LXA_4_) (1, 64). The mycobacterial component, such as Lippo Mannan from virulent MTB and *M. smeg*, induces a differential response in human MDMs. TB-LM induces higher miR-125b expression that targets the TNF mRNA and inhibits TNF biosynthesis through inhibition of TLR-2–mediated miR-155 expression, whereas *M. smeg* LM induces miR-155 expression and downregulates miR-125b and SHIP1, thereby increasing-PI3K/Akt signaling and TNF production followed by an enhanced proinflammatory response (50). The interpretation of the role of miR-155 in pro-inflammatory responses is quite contradictory as suggested by infection studies with virulent MTB and *M. smeg* LM (1, 50). This strongly reinforces the synchronized regulatory effect of miR-155 along with a host of miRNAs and, thus, cannot be studied alone (1). An inverse correlation was seen with miR-144 and TPl2 protein levels as the downregulation of miR-144 in MTB-infected human MDMs targets TPL2 mRNA, and their enhancement leads to activation of ERK1/2 phosphorylation and downstream IL-1β, IL-6, and TNF α production (65). Pro-inflammatory cytokine response is suppressed through upregulated miR-32-5p targeting Follistatin-like protein (FSTL1) (66). Downregulation of miR-365 is inversely correlated with IL-6 levels in active TB patients (67).

## miRNAs in Host Killing Machinery

The human host has an enormous killing machinery, like phagocytosis, apoptosis, and autophagy, and so on, for the invading pathogen. The intracellular MTB, however, exploits the host defense through various strategies. The recent transcriptomic approach sheds light on miRNA-based modulatory responses by Mycobacterium. The phagocytic function of macrophages is attenuated in the different stages by the Mycobacterium-induced miRNAs. The bacterial encounter and imbibe are affected through N-wasp by miR-142-3p. N-wasp is an actin-binding protein essential for actin dynamics in the phagocytosis process that was negatively regulated by upregulated miR-142-3p in J774A.1 cell line and primary human macrophages during MTB infection (68). Mononuclear cell function and phagocytosis are inhibited in active TB patients, where miR 23a-3p is downregulated. miR-23a-3p targets IRF1/SP1 through TLR4/TNF-α/TGF-β1/IL-10 signaling (69). The principal lysosomal enzyme of phagocytosis process for MTB clearance is cathepsin proteases. miR-106-5p targets the 3′ UTR cathepsin and suppresses the lysosomal activity in MTB-infected macrophages (70).

The downstream killing machinery of phagocytosed pathogen actively occurred through apoptosis of infected macrophages. Macrophages infected with Beijing strain demonstrate its virulence by escaping from host apoptosis and macrophage lysis through miR-485-3p (71). Upon infection with MTB, RAW264.7 macrophages establish attenuated apoptosis through the reduction of miR-20b-5p and elevation of its target Mcl-1 (72). Increased miR-223 expression in macrophages of active TB patients negatively suppresses forkhead box O3 (FOXO3) to inhibit apoptosis (62). The secreted protein MPT64 inhibits apoptosis of RAW264.7 macrophages *via* NF-κB/miR-21/BCl-2 pathway (73). Inhibition of apoptosis through the downregulation of Fas protein is demonstrated in THP-1 macrophages mediated by upregulated let-7b-5p (74). The decrease in the apoptotic monocytes of active TB patients and decreased apoptosis in THP-1 cells are mediated through the downregulation of FOXO-1 by miR-582-5p (75). Some of the miRNAs positively promote apoptosis for enhanced mycobacterial clearance. For example, reduction of miR-20a-5p is observed in THP-1 macrophages and CD14+ monocytes of active TB patients. Reduced miR-20a-5p inversely increases Bim expression through its target JNK2, which could promote apoptosis (76). Infection of macrophages with *M. bovis* BCG results in elevated miR-155 expression, which could induce apoptosis through PKA signaling by inhibiting PKI-*α* (77). Sp110-mediated suppression of miR-125a in RAW264.7 macrophages enhances the expression of Bmf, which could induce apoptosis (78). Upregulated miR-27b enhances p53 signaling, thus favoring apoptosis and bacterial killing by downregulating Bag2 (79).

Autophagy is a highly regulated eukaryotic cellular pathway in which intracellular pathogens are trapped in autophagosomes and degraded in lysosomes. Induction of xenophagy (a selective form of autophagy against microbes) in monocyte-derived macrophages is one of the innate immune mechanisms to intracellular pathogens, such as MTB (80). However, MTB is a successful intracellular pathogen and can escape from host responses by expression of some of the miRNAs and affects autophagy machinery (81). Certain miRNAs control both mycobacterial survival and autophagy pathways by targeting their proteins within macrophages through its altered expression (82, 83). miRNA-33 and miRNA-33* inhibit the fusion of lysosome with bacterial endosome by targeting ATG5, ATG12, LC3B, and LAMP proteins and lipid metabolism by targeting transcription factors FOXO3 and TFEB (84). The occurrence of active TB is suggested because of the suppression of autophagosome-lysosome fusion in macrophages by miR-423-5p through post-transcriptional regulation of *VPS33A* (85). Active TB patients and MTB-infected mice abundantly express miR-27a, which blocks the Ca^2+^ signaling through ER-located Ca^2+^ transporter protein CACN2D. Blockade of Ca^2+^ signaling inhibits the formation of autophagosome (86). The autophagy protein, DRAM2, promotes PtdInt3K, which initiates the nucleation of auto phagophore formation. In human and murine monocytes or macrophages, MIR144/hsa-miR-144 and miR-125a help in mycobacterial survival by forming a complex with the 3′ UTR of DRAM2 mRNA (87, 88).

TB infection triggered the expression of a new type of miRNA, i.e., miR-1958, which silences the ATG5 in RAW264.7 cells (89). miR-129-3p favors MTB survival by inhibiting ATG4B (90). miR-20a promotes BCG survival by affecting the expression of both ATG7 and ATG16L1 (91). miR-17-5p blocks autophagy by blocking ULK1 in BCG-infected RAW264.7 cells (92). Chen et al. showed that miR-30a inhibits the autophagy pathway and negative correlation between Beclin and miR-30a (93). miR-889d affects the tumor necrosis factor-like weak inducer of apoptosis (TWEAK), which maintains the granuloma formation and promotes the maturation of AMPK (94). miR-125a-5p overexpression was observed in *M. avium*–infected THP1-derived macrophages and targets STAT-3, which activates the autophagy (95). At the same time, miR-26a targets the KLF4, by which it inhibits MTB survival, and miR-17/PKCδ/STAT3 pathways also attenuate MTB by activating autophagy (96).

According to Wang et al., miR-155 targets Rheb (autophagy blocker) and promotes autophagy (97). PCED1BAS1 is down-regulated in TB patients, which directly binds with miR-155, and subsequently inhibits the activity of miR-155 (98). miR-155 expression helps in the survival of MTB by regulating ATG3 protein in dendritic cells (99). Yang et al. found that the expression of miR-155 was diminished in patients with spinal tuberculosis–induced intervertebral disc destruction and affects its target MMP-11 expression (100).

## miRNAs as Biomarkers

**Figure 2:**
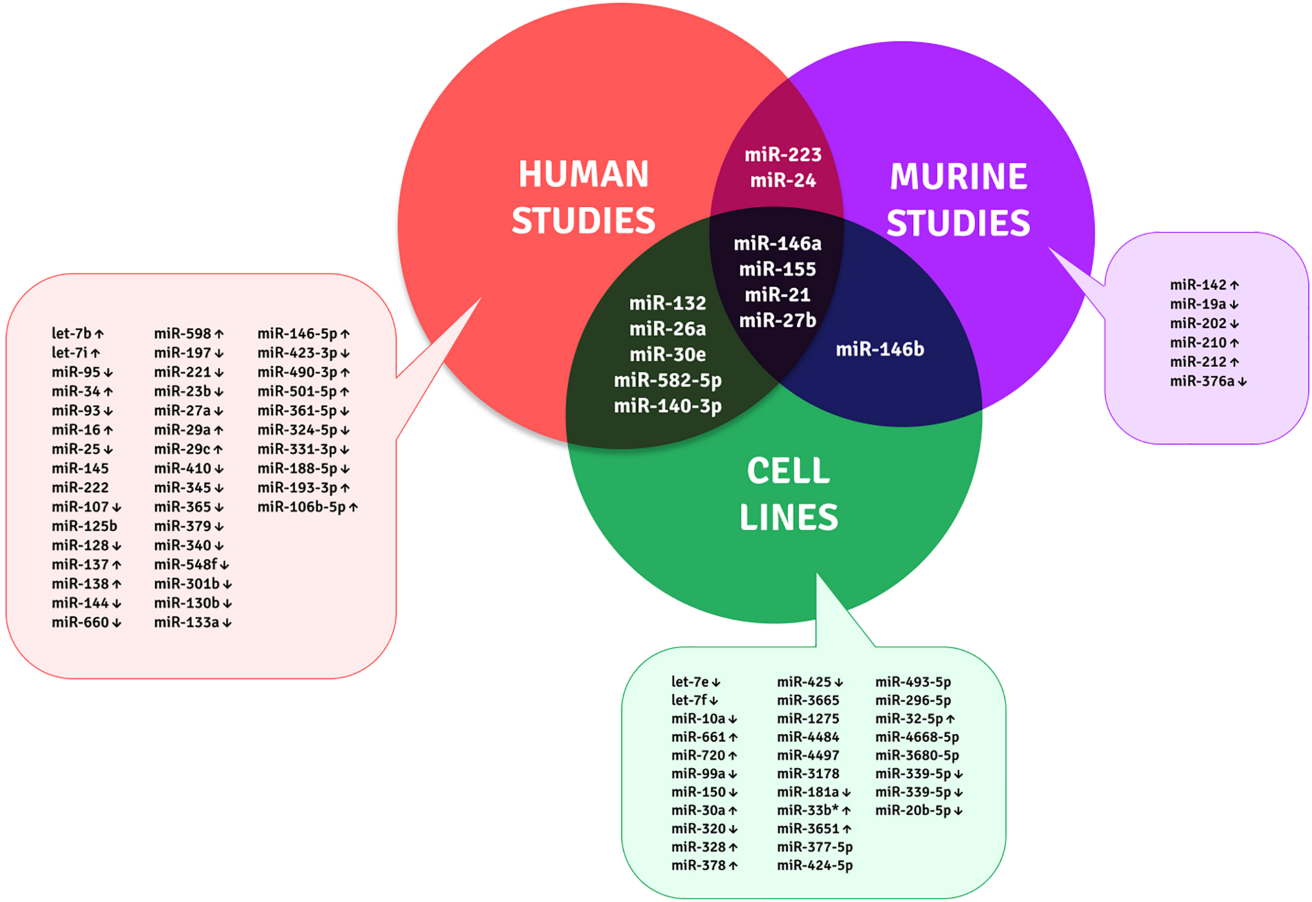
Unique and shared miRNA biomarker candidates for TB within monocytes and macrophages across human, murine, and cell line studies.

TB biomarker research is ongoing for decades as the disease still causes higher mortality due to multiple factors, such as host immune evasion by MTB, latency condition, drug resistance, and lack of prognostic and protective biomarkers. Many researchers have identified TB-specific–modulated cytokines and genes as biomarkers. However, those are not prominently emerging out since most of them are identified in smaller sample groups that lack sensitivity, differentiation ability, and reproducibility. The potent, robust, minimally invasive, rapid, universally acceptable biomarker is yet to be identified. Immune regulatory miRNAs emerge as a new class of disease-specific diagnostic markers (101, 102). The differential expression of miRNAs in disease phenomenon manifests their biomarker potential. To date, multiple studies are focused on miRNA sequencing from different samples involving PBMCs, serum/plasma, sputum, urine, and exosomes. The candidate biomarkers identified from circulation and PBMCs for discriminating TB from healthy are miR-144* (103), miR155* and miR155 (104); miR-93*, miR-3125, and miR-29a (105); miR-889, miR-576-3p, and miR-361-5p (106); miR-3179, miR-19b*, and miR-147 (11); miR-146a (107); and miR-625-3p (108). A review by Pederson et al. gives a complete biomarker profile on circulating miRNAs (109). However, our focus is on the monocyte/macrophage-based markers since most miRNAs are involved in evading their immune defense. This will help to understand the underlying pathogenesis and for identifying TB-specific biomarkers. The differential expression of miRNAs from MTB infection studies on macrophages and the monocyte-derived macrophages are depicted in **Table 1** and **Figure 2**.

**Table 1:** Monocyte/macrophage-based miRNAs as biomarker candidates for TB.

Cells	Differentially Expressed miRNAs	Analysis Platform	Reference
**Human**			
MDMs infected with MTB or BCG	miR-155, miR-146a, miR-145, miR-222, miR-27a, and miR-27b	Taqman low-density array	(110)
MDMs from TB patients, LTB, and Healthy individuals	TB *vs* HC:	Taqman microarray quantitative PCR	(71)
	Upregulated (hsa-miR-16, hsa-miR-137, hsa-miR-140-3p, hsa- miR-193a-3p, hsa-miR-501-5p, and hsa-miR-598)		
	Downregulated (hsa-miR-95)		
	LTB *vs* TB: Upregulated (hsa-miR-101 and hsa-miR-150)		
	Unique expression in LTB (miR-146b-3p and hsa-miR-296-5p)		
MDM infected with TB LM	miR-125-b	qPCR	(50)
MDM infected with *M. smeg* LM	miR-155		
MDM infected with MTB H37Rv	Upregulated (miR-155, miR-21, miR-146a, miR-29a, miR-26a, let-7b, miR-34, miR-132 & miR-138)	Nanostring nCounter miRNA assay	(65)
	Downregulated (miR-660, miR-144, miR-301b, miR-128, miR-423-3p, miR-410, miR-27a, miR-93, miR-107, miR-345, miR-221, miR-25, miR-23b, miR-361-5p, miR-130b & miR-340)		
MDM infected with MTB	Upregulated (miR-132, miR-146-5p, miR-30e, let-7i, miR-490-3p, miR-29c, miR-26a, miR-21, let-7b & miR-29a)	Nanostring nCounter miRNA assay	(57)
	Downregulated (miR-25, miR-23b, miR-331-3p, miR-423-3p, miR-548f, miR-340, miR-24, miR-107, miR-93, miR-324-5p, miR-188-5p, miR-130b, miR-410, miR-361-5p, miR-197, mir-27a, miR-128, miR-345, miR-379, miR-133a & miR-221.		
Primary monocytes and MDMs from active TB patients and controls	Upregulated-miR-582-5p	qPCR	(75)
Primary macrophages from TB patients *vs* controls	Upregulated miR-223	qPCR	(62)
Macrophages from TB patients and controls	Downregulated miR-365	qPCR	(67)
MDM infected with MTB	Upregulated miR-106b-5p	qPCR	(70)
**Mouse**			
BMDMs infected with MTB	6 upregulated (miR-21, miR-21*, miR-146a, miR-146 b, miR210, and miR-155), 1 downregulated (miR-223)	Microarray and qPCR	(111)
BMDMs infected with Mtb	4 upregulated (miR-24, miR-142, miR-155, and miR-212) and 3 downregulated (miR-19a, miR-202, and miR-376a)	Gene expression microarray	(112)
BMDMs infected with BCG	miR-21	Taqman quantitative real-time PCR	(63)
BMDMs infected with MTB	Upregulated miR-27b	qPCR	(79)
BMDMs infected with MTB	3 upregulated (miR-155, miR-146a & miR-21)	Taqman low-density arrays	(1)
Mouse peritoneal macrophages & BMDMs	Upregulated miR-146a	qPCR	(60)
**Cell Line**			
U937 macrophages	149 DE (miR-424–5p, miR-493-5p, miR-27 b-3p, miR-296-5p, miR-377–5p, miR-3680–5p)	Microarray	(113)
THP-1 cells infected with Beijing/W or non-Beijing/W strains	13 downregulated (let-7e, let-7f, miR-10a, miR-21, miR-26a, miR-99a, miR-140–3p, miR-150, miR-181a, miR-320, miR-339–5p, miR-425, and miR-582-5p)	Taqman microarray quantitative PCR	(71)
THP-1 cells infected with virulent or avirulent Mtb strains	9 DE (miR-30a, miR-30e, miR-155, miR-1275, miR-3665, miR-3178, miR-4484, miR-4668-5p, and miR- 4497)	Microarray	(114)
THP-1 cells infected with MTB HN878	12 upregulated (miR-33b*, miR-146a, miR-155, miR-132, miR-146b-5p, miR-720, miR-30e, miR-661, miR-140-3p, miR-3651, miR-328, and miR-378		(115)
THP-1 cells and U937 cells	Upregulated miR-32-5p	qPCR	(66)
THP-1 cells	Upregulated miR-30a	qPCR	(55)
RAW264.7 cells and infected with MTB	3 upregulated (miR-155, miR-146a, and miR-21)	Taqman low-density arrays	(1)
RAW264.7 cells infected with MTB	Upregulated miR-27b	qPCR	(79)
RAW264.7 cells infected with MTB	Downregulated let-7f	SYBR Green-based miRNA profiling array	(58)
RAW264.7 cells	Downregulated miR-20b-5p	Semi quantitative PCR	(72)

Although many studies are available on the macrophage infection-derived miRNAs, the actual *in vivo* scenario of a patient is minimal. The limitations of these biomarker candidates are variable between the studies, and each was performed on identifying the miRNA targets for understanding the disease pathology. In the future, the biomarker efficiency of these candidates should be largely examined as multi-centric studies with diverse ethnicities.

## miRNAs in Host-Directed Therapy (HDT)

Host-directed therapy is one of the emerging strategies to improve the host immunity and eliminate pathogens in which vitamins, repurposed drugs, cytokines, miRNAs, and, monoclonal antibodies are used as an adjunct with chemotherapy. It helps to control challenges of TB treatments, such as drug resistance, the toxicity of chemotherapy, and immune reconstitute inflammatory syndrome, and so on (116). Induction of autophagy is one of the host-mediated therapy for tuberculosis (117) and is induced by mTOR kinase inhibitors and certain immunomodulators, such as rapamycin and vitamin D_3_, respectively (118, 119). The PubMed search on miRNAs in HDT for tuberculosis yielded no results. However, many HDT strategies using miRNAs have been proposed by Sabir et al. (96). They suggested direct administration of miRNAs or the use of siRNAs to modulate the host responses. The downregulated anti-mycobacterial miRNAs can be induced by synthetic oligos, and the overexpressed pro-mycobacterial miRNAs can be repressed using anti-miRNA complementary to mature miRNA (120–122). This approach will benefit the host in achieving the proper signaling and their downstream pro-inflammatory responses. Synthetic delivery of miRNAs to macrophages is possible with nanoparticles or liposomes (123, 124). Novel HDT approaches on miRNA-mediated induction of host killing machinery (phagocytosis, apoptosis, and autophagy) could be a beneficial therapy to evade the pathogen strategies and for efficient pathogen clearance.

## Future Perspectives

The research of miRNA-mediated regulation of TB is enormous; however, the pro diagnosis and effective therapy for TB are lacking widely. As miRNAs are regulators and modulators of the immune response, the avenue for potential biomarkers and therapeutic possibilities are much promising. Some of the key factors to be considered for future research on miRNA are as follows:

1. Various circulating miRNAs are available from many studies as biomarkers but research on identifying cell-oriented miRNAs, particularly monocytes and macrophages will help better to understand the evasion of initial defense.
2. Research on identified miRNAs to investigate their diagnostic efficacy and therapeutic value is highly needed. This will help address whether this differential expression is really specific for TB or overlaps with a disease of similar pathology.
3. The mycobacterial strain-specific miRNA expression is another concern since there is diversity in TB strains, and the distribution is different in different geographical locations.
4. Deep single-cell sequencing approach may enable the complete miRNA profile for better understanding their bio-signatures.
5. Patient samples from all disease stages of TB at diagnosis and during treatment may give the disease-based profile during the entire course of infection for understanding their pathophysiology.
6. Novel HDT approaches using nanoparticle and siRNAs for direct modulation of these expression signatures to induce the host-mediated defense responses against Mycobacterium will open up a better therapy adjunct with minimal chemotherapy.
7. More animal studies with miRNA/long non-coding RNA intervention for TB therapeutics should be carried out and explored.

Future collaborative efforts involving multidisciplinary approach in various ethnic population with multiple factors (age, gender, mycobacterial strain, disease stage, other chronic lung infections, and inflammatory disease criteria) on these short miRNAs from body fluids and cells could predict the valuable miRNA biosignature network for biomarker discovery and host-directed therapy.

## Author Contributions

PS and KP contributed to the literature collection, writing, drafting, and revision of the manuscript. PS, UR, and RB participated in the conception of the idea, design, drafting, revision, and approval of the manuscript. All authors contributed to the article and approved the submitted version.

## Funding

PS has been supported by the DST-INSPIRE fellowship. KP has been supported by the ICMR Fellowship. RB has been supported by the DBT Ramalingaswami Fellowship, Ministry of Science and Technology, Government of India.

## Conflict of Interest

The authors declare that the research was conducted in the absence of any commercial or financial relationships that could be construed as a potential conflict of interest.
